# Revisiting clinico-epidemiological pattern of human rickettsial infections in the central region of Sri Lanka: a hospital based descriptive study

**DOI:** 10.1186/s13104-017-2727-1

**Published:** 2017-08-11

**Authors:** Kosala Gayan Weerakoon, Senanayake A. M. Kularatne, Jayanthe Rajapakse, Sanjaya Adikari, Kanchana Udayawarna

**Affiliations:** 1 Department of Parasitology, Faculty of Medicine and Allied Sciences, Rajarata University of Sri Lanka, Saliyapura, Sri Lanka; 2 Department of Medicine, Faculty of Medicine, University of Peradeniya, Peradeniya, Sri Lanka; 3 Department of Veterinary Pathobiology, Faculty of Veterinary Medicine, University of Peradeniya, Peradeniya, Sri Lanka; 4 Department of Anatomy, Faculty of Medicine, University of Peradeniya, Peradeniya, Sri Lanka

**Keywords:** Rickettsial infections, Spotted fever group, Clinico-epidemiology, Sri Lanka

## Abstract

**Background:**

This study revisits the clinico-epidemiology and serological patterns of rickettsioses in the central region of Sri Lanka and highlights the need of advanced diagnostics for precise identification of species responsible for rickettsioses.

**Methods:**

The patients treated for rickettsioses between November 2009 and October 2011 were recruited for the study from Teaching Hospital, Peradeniya. Clinical characteristics and serology results were used for diagnosis.

**Results:**

Study included 210 patients (mean age 44 years ± 3.2) and of them 188 (90%) had positive IgG and/or IgM sero-reactivity for spotted fever group (SFG). Of them, 134 had IgG titre ≥1/256 for SFG and presented with fever and skin rash. They also had headache [*n* = 119 (89%)], myalgia [*n* = 103 (77%)], arthralgia [*n* = 89 (66%)] of large joints, conjunctival injections [*n* = 83 (62%)], thrombocytopenia (*n* = 78.58%), anaemia (*n* = 14.10%), leukocytosis [*n* = 35 (26%)], leucopenia [*n* = 17 (13%)], elevated aspartate transaminase [*n* = 69 (52%)] and alanine transaminase [*n* = 73 (55%)].

**Conclusions:**

Predominance of SFG rickettsioses are reiterated, possibly transmitted by ticks. Joint disease is common with occasional fern leaf skin necrosis. Changing socio-economic conditions, vegetations, contact with domestic and wild animals, abundance of vectors would have contributed for emergence and sustenance of SFG in the region. Further research is needed to identify the causative agents and the mode of transmission.

## Background

Rickettsial infections have re-emerged in Sri Lanka with predominance of spotted fever group (SFG) in the central hills [1–8]. Even though, clinical picture of the infection is typical in many situations, variations have been reported [1, 2, 4]. The disease is prevalent mainly in rural or suburban areas of the country where the middle or lower socioeconomic classes are at risk of exposure to wild and domestic animals. The occupations involving outdoor activities and exposure to arthropods are predominantly seen among the patients [1, 3, 6]. Still wide gulfs of knowledge exist in areas such as identification of vectors, mode of transmission, reservoirs and the rickettsial agents in the country.

The western slope of the hilly terrain of the Central Province of the island shows heavy prevalence of SFG rickettsioses and occasional detection of typhus group (TG) and scrub typhus (ST) [1, 6, 9]. Variations of epidemiology and the clinical manifestations of SFG have been observed over a decade, and currently available serological and molecular diagnostics have widened the scope for further studies. Being a treatable infection, improvement of clinical awareness by studying the current trends of the disease is essential. This is feasible only by doing uninterrupted surveillance of the disease and reporting the clinical picture with its variations.

So far the confirmation of the diagnosis of rickettsioses has been based on serological evidence during acute and/or convalescent phase of the illness [2, 3, 6]. Currently available indirect immunofluorescence (IFA) assays offer only a limited selection of antigens that cross-react with different rickettsiae in the country [10]. It is possible that IFA would be sufficient in diagnosing rickettsioses but it lacks specificity to diagnose different aetiological agents in Sri Lanka. This study revisits the rickettsial infections in the Central Province of Sri Lanka, focusing on clinico-epidemiological and serological patterns in the region. The study also highlights the need of advanced diagnostics for precise identification of species of rickettsioses and to find out mode of transmission locally.

## Methods

### Study population, sample selection and data collection

All patients admitted to the Medical Unit at Teaching Hospital, Peradeniya, Sri Lanka during the period from November 2009 to October 2011 with any two of the following clinical criteria were included in the study; presence of fever (body temperature >38 °C) for more than 5 days, associated macular, papular or maculopapular discrete skin rash and rapid defervescence with doxycycline or chloramphenicol [1]. These three clinical parameters have been used as clinical characteristics common to rickettsial infections in previous studies [1, 2, 10]. Patients were interviewed, examined and followed up during the hospital stay and after discharge. All clinical, demographic and epidemiological details were recorded on individual formatted data sheets.

### Collection of specimen

Two ml of blood was drawn on 5th to 7th day of illness into plain tubes and similarly the convalescent samples were collected 2–3 weeks after being discharged from the hospital. Serum was separated and stored in −20 °C until further tested using IFA.

### Serological testing

Serological testing was based on IFA established in a local laboratory. Specific rickettsial antigens were obtained from WHO Reference center for Rickettsial & Bartonella Associated diseases, CDC, Atlanta, USA. Frozen lyophilized antigen pellets of *Rickettsia conorii* (Strain Malish) of SFG, *Orientia tsutsugamushi* (Strain Karp) of ST and *Rickettsia typhi* (Strain Wilmington) of TG in Vero cells were used for testing. One antigen vial was dissolved with 0.5 ml of PBS and a 10 µl drop of the solution was then put into each well of an IFA slide, pre-coated with 1% BSA. Once the antigen was dried the slide was fixed with acetone and then stored in −20 °C until further use. Antibodies were detected using fluorescein conjugated goat anti-human IgG and IgM. IFA testing was considered sero-positive with a minimum titre value of 1/32, for both IgG and IgM. Plasma samples demonstrating positive serological reactions to IgG were further tested to obtain individual end point titres. The final diagnosis of rickettsial infection was defined on the basis of their clinical criteria and the presence of IFA IgG titre ≥1/256 and IgM positivity. Previous evidence on serological diagnostic values were used to adopt a set of criteria for defining the group of patients with a maximum possible accuracy in the considered practical setting amidst the logistical constrains [1, 3, 11, 12].

### Data processing and statistical analysis

Data gathered in the data sheets was processed and individual data points were stored in a computerized data base (Excel, Microsoft). The data file was rechecked with the data sheet to ensure correct entry of data. Basic descriptive analyses were done by using the measures of central tendency and the data were analysis was done with Minitab, version 14.0, Minitab Inc. USA.

### Definitions

#### Rapid defervescence

Dramatic subsiding of fever within 2 days.

#### Thrombocytopenia

Platelet count <150 × 10^6^/l.

#### Leukocytosis

White blood cell count >11 × 10^6^/l.

#### Leukopaenia

White blood cell count <4 × 10^6^/l [13].

#### Elevated liver transaminases

Alanine (ALT) and aspartate (AST) transaminases >40 IU/l [14].

#### Anaemia

Normal range of haemoglobin level for males is 13.5–17.7 g/dl and that of females is 11.5–15.5 g/dl. However, no exact cutoff values for Sri Lankan population are available. Therefore, a haemoglobin level less than 11 g/dl was considered as a safe margin to detect anaemia, which could possibly be attributed to the infection.

#### Age categories

Subjects in this study were categorized into three age groups in the final analysis: young (<30 years), middle aged (30–60 years) and elderly (>60 years).

#### Animal contacts

Direct handling of animals or sharing common environmental areas of animal roaming during day to day activities. Especially the latter was considered in relation to contact with wild animals.

## Results

### General description

A total of 210 patients were included for serological assessment comprising of 107 (51%) males and 103 (49%) females. Details of the age and gender distribution of the 134 patients who had high titre of IgG for SFG are summarized in Table 1.

**Table 1:** Age and gender distribution of the SFG patients

Parameter	SFG patients (*n* = 134)
Age in years, mean (95% confidence limits)	44 (±3.2)
Age categories comparison, *n* (% and 95% confidence limits)	
Elderly	32 (24 ± 7.2)
Middle age	64 (48 ± 8.5)
Young	38 (28 ± 7.6)
Gender distribution, *n* (% and 95% confidence limits)	
Male	69 (51 ± 8.5)
Female	65 (49 ± 8.5)

### Serology

Results of serological testing with IFA are summarized in Table 2. Of the total, 188 (90%) had positive IgG and/or IgM sero-reactivity for SFG and one patient in this group was positive for TG as well (Table 2). Twenty-two (10%) were negative for both IgG and IgM in all three categories. Of the SFG sero-positive group, 134 had both IgG titre ≥1/256 and positive IgM titres and were defined as SFG patients. This group was considered in the final analysis of epidemiological, clinical and investigation parameters.

**Table 2:** Distribution of patients according to the IgG antibody titre levels in acute sera

IgG antibody titer levels to *R. conorii* antigen	Number of patients (% and 95% confidence limits)^a^
<1/32	22 (10.5 ± 4.2)
1/32	16 (07.6 ± 3.6)
1/64	17 (08.1 ± 3.7)
1/128	11 (05.2 ± 3)
1/256	70 (33.3 ± 6.4)^b^
1/512	53 (25.2 ± 5.9)
1/1024	16 (07.6 ± 3.6)
1/2048	04 (01.9 ± 1.9)
1/4096	01 (0.5 ± 0.9)
Total	210 (100)

^a^Percentages were calculated, out of the total number of 210 patients

^b^One patient had mixed seroreactivity to *Orientia tsutsugamushi* antigen with a similar titre

### Epidemiological factors

**Fig. 1:**
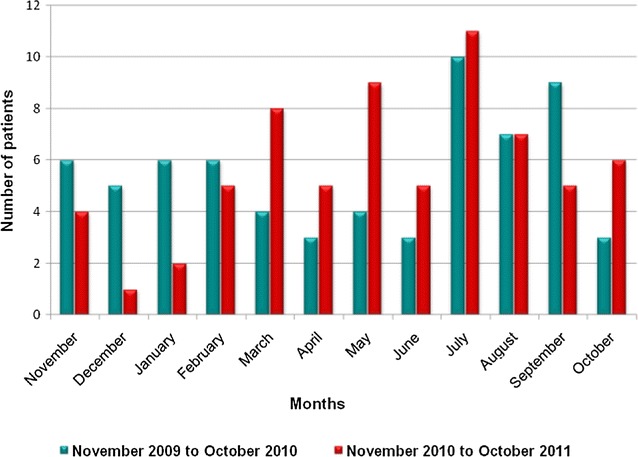
Monthly distribution of the cases over the 2 years of the study (*n* = 134)

**Fig. 2:**
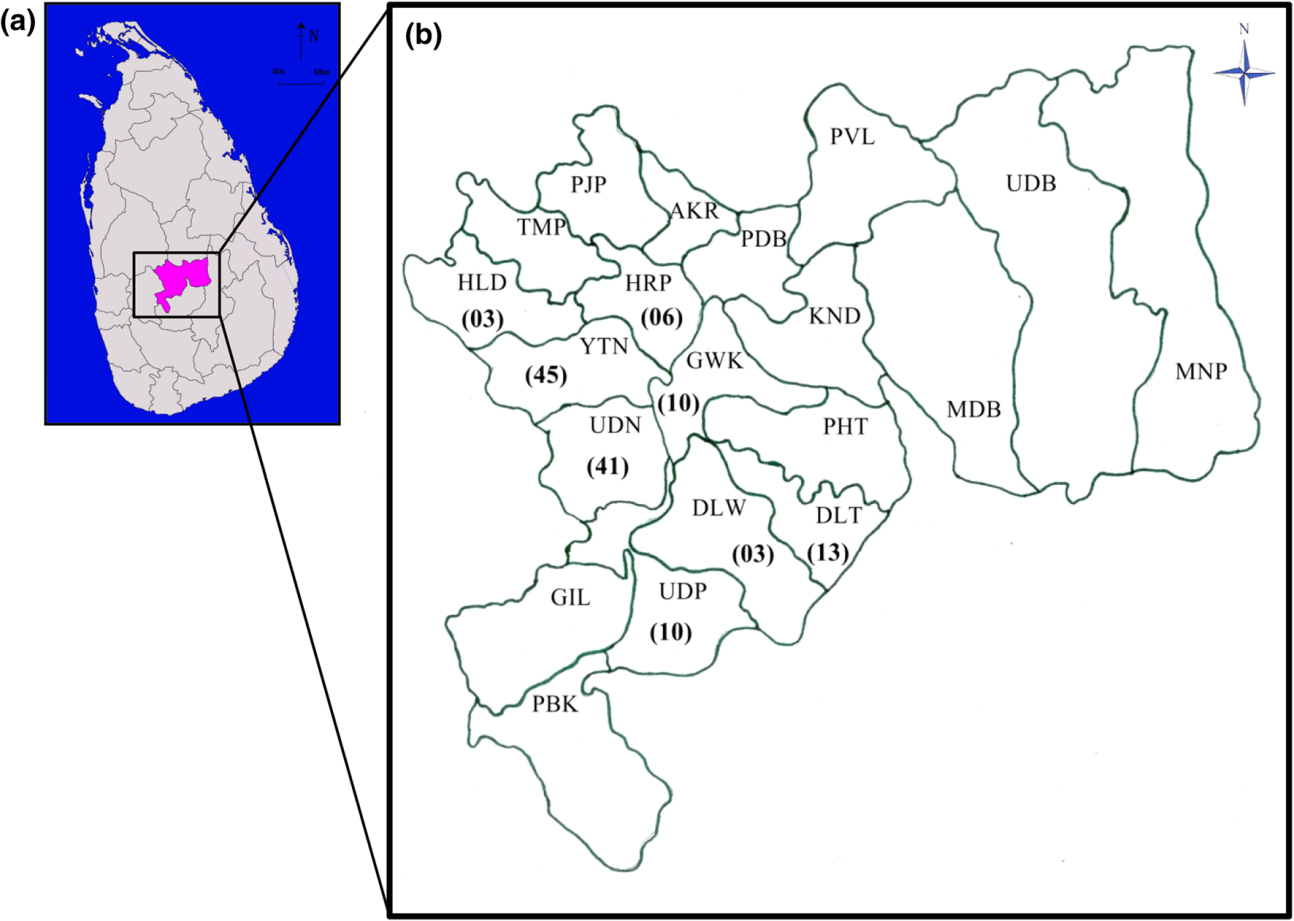
Geographical distribution of SFG patients. **a** Map of Sri Lanka highlighting the Kandy district. **b** Map of Kandy district locating DS areas. Number of patients presented from each DS is given within parenthesis. Abbreviated names of DSs; *AKR* akurana, *DLW* doluwa, *DLT* delthota, *GIL* gangaihalakorale, *GWK* gangawatakorale, *HLD* hatharaliyadda, *HRP* harispaththuwa, *KND* kundasale, *MNP* minipe, *MDB* medadumbara, *PBK* pasbagekorale, *PDB* pathadumbara, *PHT* pathahewaheta, *PJP* poojapitiya, *PVL* panvila, *TMP* thumpane, *UDB* ududumbara, *UDN* udunuwara, *UDP* udapalatha, *YTN* yatinuwara (modified from the original source: Annual Central Province Health Bulletin, 2007 and the map of Sri Lanka was obtained from the open web source: http://commons.wikimedia.org/wiki/File:Sri_Lanka_districts_Kandy.svg)

Epidemiology of SFG patients (*n* = 134) showed distribution of cases throughout the year with peaks in July and August (Fig. 1). Majority of the patients were residents in the divisional secretariats (DS) of Udunuwara, Yatinuwara, Delthota, Gangawatakorale and Uda palatha of Kandy district (Fig. 2). These regions belong to the hill country wet zone of the island situated about 900 m above the sea level consisting semi-urban to rural village settings interspersed with high grown evergreen vegetation. The inter-monsoon and south-western monsoon brings average annual rain fall above 2600 mm and the daytime temperature is about 29 °C (range 20–31 °C). The houses of the patients were solidly constructed permanent houses with asbestos [*n* = 101 (75%)] and tiled roofing [*n* = 24 (18%)] and cemented/tiled floor 131 (98%). Majority of the houses were surrounded by coverings of thick vegetations with scrub plantations [*n* = 64 (48%)] and/or abandoned areas [*n* = 31 (23%)].

In the occupation categories, 25 (19%) of the group were field or outdoor workers and 21 (16%) were office or indoor workers. Ten (7%) were students and 44 (33%) were house wives. The rest, 34 (25%) were either unemployed or retired workers. Despite their regular occupation all had part time involvement in outdoor work. Considering the domestic and wild animal contacts, 99 (74%) of the group kept pet animals such as dogs and/or cats. Only a minority of them [*n* = 18 (17%)] were caged or chained while the majority were left free. Eight (6%) were involved in animal farming, which included hens, goats and cattle. Ecto-parasites were noted among the pet and farm animals [*n* = 101 (75%)], particularly ticks. Of these, only in 20 (15%) cases, the parasite control strategies had been used. Both manual removal and chemical control had been used periodically. Seventy (52%) had contact with wild animals including wild boar in 67 (50%) patients. Civet cats and monkeys were the other identified animal contacts. Other than these animal contacts, rodent sites were noted around human dwellings in 92 (69%) cases. Considering the human behaviour in relation to the exposure to the vector, 18 (13%) were routinely involved in wood picking around shrubs and 40 (30%) were regularly involving in outdoor and garden activities. Nineteen (14%) of the patients had a recent history of a tick and flea bites. Commonest bite sites were hands and legs [*n* = 13 (68%)], while in some cases it was either the neck [*n* = 4 (21%)] or the ear [*n* = 2 (11%)].

### Clinical characteristics

**Fig. 3:**
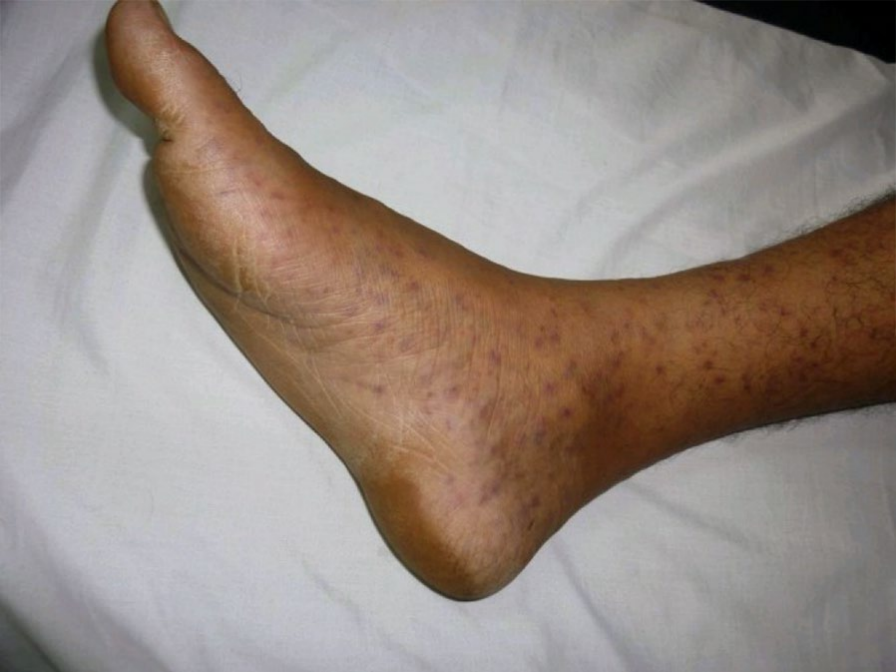
Discrete erythematous maculopapular skin rash in a lower limb

Major clinical characteristics of the SFG patients are summarized in Tables 3 and 4. All patients in this group had fever and skin rash. Fever was basically of abrupt onset, high grade and intermittent. Majority [132 (98%)] had maculopapular skin rash, mainly of erythematous colour (Fig. 3) with a few [8 (6%)] having necrotic rash [15]. Average duration of fever on admission was 6 days (range 2–21 days) and that of skin rash was 2 days (ranging from 1–7 days). Of this group, 119 (89%) had experienced skin rash following the onset of fever and average duration of the time gap between the appearance of these two symptoms was 3 days. Rest of the 15 (11%) patients had noticed fever and skin rash together. Seventeen of the total group had neurological manifestations. Of them, high titre SFG positive group had, neck stiffness [5 (4%)], confusion [7 (5%)], extrapyramidal rigidity [9 (7%)], tremors [8 (6%)] and motor weakness of limbs [2 (1.5%)] [16]. A proportion of the group had [89 (66%)] arthralgia. Involvement of the joints among the patients who had arthralgia is given in Table 4. Three joint categories, namely large joints (ankle, knee, wrist, elbow and shoulder), small joints (joints of hands and feet) and joints of the axial skeleton (neck, back and hip) were compared and the involvement of large joints was predominant (Table 4). Among the SFG positive patients, single joint involvement was noted in 12 (9%) patients. Seventy-seven (58%) had the involvement of more than one joint and of them 15 (19%) had all the joints involved. Sixteen (12%) patients who had ankle joint pain had mild swelling with cutaneous oedema and tenderness suggesting arthritis. All these 16 patients were over 60 years of age (range 60–84 years).

**Table 3:** Main clinical features of the SFG patients

Clinical features	Number of patients, *n* = 134
Symptoms [number of patients: *n* (% and 95% confidence limits)]	
Fever	134
Skin rash	134
Headache	119 (89 ± 5.3)
Myalgia	103 (77 ± 7.1)
Nausea	83 (62 ± 8.2)
Arthralgia	89 (66 ± 8)
Vomiting	54 (40 ± 8.3)
Cough	37 (28 ± 7.6)
Abdominal pain	19 (14 ± 5.9)
Diarrhoea	19 (14 ± 5.9)
Signs [number of patients: n (%)]	
Conjunctival injections	83 (62 ± 8.2)
Hepatomegaly	22 (16 ± 6.2)
Crepitations in the lungs	17 (13 ± 5.7)
Pallor	14 (10 ± 5.1)
Lymphadenopathy	08 (06 ± 4)
Possible tick bite marks	08 (06 ± 4)
Cardiovascular parameters (mean, 95% confidence limits)	
Pulse/min	85 (±1.9)
Systolic blood pressure in mmHg	115 (±2.9)
Diastolic blood pressure in mmHg	75 (±1.6)

**Table 4:** Involvement of joints in the SFG patients

Joint/Joint category involved^a^	Number of patients, *n* = 134
Joint involved [n (% and 95% confidence limits)]	
Ankle	73 (54 ± 8.4)
Knee	71 (53 ± 8.4)
Elbow	64 (48 ± 8.5)
Wrist	47 (35 ± 8.1)
Shoulder	46 (34 ± 8)
Hand	43 (32 ± 7.9)
Lower back	40 (30 ± 7.8)
Feet	39 (29 ± 7.7)
Neck	20 (15 ± 6.1)
Joint category involved [n (% and 95% confidence limits)]	
Large joints	85 (63 ± 8.2)
Small joints	44 (33 ± 8)
Joints of axial skeleton	40 (30 ± 7.8)

^a^Listed in the descending order of the frequency

### Routine laboratory investigation findings

Results of the basic haematological tests and the abnormal findings of these investigations are summarized in Table 5. Anaemia, thrombocytopenia, leukocytosis as well as leukopaenia and elevated liver transaminases were among the main abnormalities.

**Table 5:** Basic laboratory investigation findings of SFG patients

Investigation findings and abnormalities detected	Results, *n* = 134
Basic investigation findings (mean, 95% confidence limits)	
Haemoglobin, g/dl	12 (±0.2)
Platelet count ×10^6^/l	158 (±12.9)
Leukocyte count ×10^6^/l	09 (±0.7)
Neutrophils differential count, %	69 (±1.9)
Lymphocytes differential count, %	24 (±1.7)
Aspartate aminotransferase (AST), U/l	69 (±9.7)
Alanine aminotransferase (ALT), U/l	73 (±9.7)
Abnormalities detected in routine investigations (n, % and 95% confidence limits)	
Anaemia (haemoglobin % <11 g/dl)	14 (10 ± 5.1)
Thrombocytopenia	78 (58 ± 8.4)
Leukocytosis	35 (26 ± 7.4)
Leukopaenia	17 (13 ± 5.7)
AST > 40 U/l	62 (46 ± 8.4)
ALT > 40 U/l	64 (48 ± 8.4)

### Treatment and outcome

Of the SFG patients, 98 (73%) were treated with doxycycline alone, 23 (17%) were treated with chloramphenicol alone and both drugs were given together to the remaining 13 (10%) patients. Of the total of 210 patients, two patients (47 and 62 year old males) who did not belong to the high antibody titre category, had neurological complications and died [16]. In the rest of the group, a complete defervescence was noted in 1–3 days of commencement of the antibiotic treatment. Nevertheless, 29 of the whole group (21 of the SFG patients) were administered intravenous hydrocortisone, along with antibiotics as their clinical parameters were initially deteriorating. These included deterioration of general well being, development of skin necrosis, thrombocytopenia, neurological complications and elevated hepatic transaminases. This combined therapy might have facilitated the improvement in clinical parameters and the recovery of these patients.

## Discussion

Our revisit found predominance of SFG in the region and identified different related epidemiological factors. Ninety per cent of clinically suspected cases of rickettsial infection had positive sero-reactivity for SFG in the series based on clinical criteria that had been previously established [1, 2, 17]. Although antigens of three different species (SFG, TG and ST) are used for serological testing, they are known to cross react with antibodies related to rickettsial species from any part of the world. Thus, it is important to use antigens obtained from locally isolated rickettsial agents to catch all definitive cases. Current reports on the emergence of many new rickettsial species from all over the world would demand such need [18].

Examination of serum samples of acute illness and the paired samples obtained 2–3 weeks later to demonstrate a rising antibody titer are the approaches to confirm the serodiagnosis [19]. However, in the present study, paired sera were tested only in six patients due to practical constraints and they showed fourfold rise in the antibody titers between acute and convalescent serum samples. Majority of patients did not visit the clinic after discharging from the hospital for paired sera testing. Similar practical difficulties have been encountered in previous studies indicating a need of alternative methods [1, 2]. Moreover, serological evidence with high antibody titres in acute serum samples alone has been considered confirmative in certain previous studies [3, 11], Premaratne et al. in 2012 [12], attempted to determine a clinically helpful diagnostic algorithm and showed, if the acute serum sample was obtained following 7 days of the illness, a single IgG titre of 1/256 can be considered diagnostic, with the presence of supportive clinical evidence. In this background, the diagnosis of rickettsial infection in the present study was defined on the basis of having clinical criteria and the presence of a specific combination of IFA, IgM and IgG titres in acute sera.

All cases in the study group qualified for the serodiagnosis of SFG rickettsioses, because they were seroreactive only against *Rickettsia conorii* antigen. Further, one of them showed seroreactivity against *Orientia tsutsugamushi* antigen as well. Of the three antigenic categories, cross reactions between SFG and TG have been reported [20]. Nevertheless, cross reactivity between SFG and ST has not been reported earlier. Therefore, the single case which showed reactivity to both antigens could be a result of either past exposure or co-infections by the two.

Findings of this study are consistent with the previous reports that showed predominance of SFG in the region [1, 3, 6, 9] whilst predominance of ST has been reported in the Western Province which belongs to low country wet zone of Sri Lanka. Moreover, it is recognized that the geographical sero-prevalence of rickettsioses is not homogenous within a country. Rate of prevalence differ from one area to another due to the different epidemiologic and climatic environments [21]. However, factors such as availability of host species and habitats could also add to differences observed. Differences in the virulence of the local species can also lead to some differences in the sero-positivity among different areas [22].

In a clinical setting, the rickettsial infections should come as a differential diagnosis of febrile illnesses such as dengue, leptospirosis and chikungunya. Diagnosis of rickettsial infection is rewarding as it is treatable with anti-rickettsial antibiotics, where recovery is dramatic and complete. Although rapid improvement with doxycycline is compatible with a rickettsiosis, other infections can also respond to doxycycline (e.g., mycoplasma infection, Q fever, leptospirosis) and may be difficult to exclude during early illness. However, in this study the diagnosis of a rickettsial infection was not solely based on response to antibiotic therapy, but also with other clinical and serological criteria as defined above.

At the same time, missing the diagnosis or delayed diagnosis may lead to catastrophes. Therefore, it is imperative to understand the whole clinical spectrum of the disease including atypical presentations. To address this issue, the current study describes the clinical spectrum of the rickettsial infections in details. A wider range of general clinical manifestations were noted. Joint involvement was common in this cohort of patients and it highlights the importance of joint diseases as a clue to the clinical diagnosis when the presentations are atypical. Important neurological manifestations noted in this group of patients were published separately highlighting their diagnostic importance [16]. It is noteworthy that the atypical presentations of rickettsioses are not uncommon and those could bring about considerable hospital morbidity and mortality which are currently considered as due to unknown causes or due to old age. Thus considerable degree of suspicion is needed in an endemic setting to detect atypical presentations.

Most of the patients in this cohort had contacts with pet, farm or wild animals. More than half the group who had contacts with wild animals had contacts of wild boar while civet cats and the monkeys were the other contacts. In addition to large animals, majority of the patients had contacts with rodents as well. The vectors and reservoirs of SFG rickettsioses in the island are not yet known, but hard ticks could be the vector similar to other regions of the world [23]. Ticks may also act as reservoirs of rickettsiae whereas the role of vertebrates as reservoirs of rickettsiae is yet to be determined [24]. Ticks spend most of their lives in the vegetation and soil and make only brief appearances on their hosts to feed and mate. Thus, the vegetation is not only the main habitat of the ticks but also an important factor that determines the distribution.

Some recognized vectors of SFG rickettsioses are *Rhipicephalus sanguineus*, *Haemaphysalis* and *Dermacentor* species, especially in the Asian regions [25]. Canine studies have shown higher sero-positivity rates in dogs compared to humans in endemic zones from where the disease has been reported [26]. This is due to the fact that movement of dogs in vector habitats such as jungles or abandoned areas are higher than that of man [27]. A recent study in the central region of Sri Lanka in dogs had shown high serological reactivity against three rickettsial species indicating dogs role as a reservoir in the rickettsial infections [28].

The current study surveyed epidemiological factors possibly related to the rickettsioses in the central hills of Sri Lanka. It is likely that the prevailing environmental conditions, reservoir animals, vectors and vector-human contacts are more conducive for the spread of SFG in the central hills. Either the brown dog tick or ticks in the wild animals such as wild boar could be the possible vectors of SFG in the study region. A proportion of patients recalled of having prior tick bite and roaming of wild animals in the home gardens. However, this link needs scientific proof. There could be more than one tick species and reservoir animals involved in human rickettsioses. Finding out confirmatory evidence for all these possibilities are dependent on and the exact identification of definitive species causing the rickettsioses in the region. This needs the use advanced molecular diagnostics in future and it also would be helpful to improve the serological diagnostics with the use of antigens of local strains. This knowledge is very important in prevention and spread of infection and it paves the way for future research. The findings of this study could be the foundation of future clinical and molecular studies of rickettsioses in Sri Lanka to identify specific rickettsial species, reservoirs, vectors and the mode of transmission.

## Conclusions

Predominance of SFG rickettsioses in the central region of, Sri Lanka are reiterated in this clinico-epidemiological revisit. A wider range of general clinical manifestations were noted. Joint involvement is a common with occasional fern leaf skin necrosis, highlighting the importance of such manifestations as clues to the clinical diagnosis in atypical presentations. Moreover, it is likely that the prevailing environmental conditions, reservoir animals, vectors and vector-human contact are more conducive for the spread of SFG in the central hills and is possibly transmitted by ticks. Further research and advanced diagnostics are needed to identify the causative species and the mode of transmission to implement preventive strategies.

## Abbreviations

ALT: alanine transaminase
AST: aspartate transaminase
IFA: indirect immunofluorescence assay
SFG: spotted fever group
ST: scrub typhus
TG: typhus group
